# Impact of fatigue on nursing care rationing in paediatric haematology and oncology departments – a cross-sectional study

**DOI:** 10.1186/s12912-021-00663-7

**Published:** 2021-08-10

**Authors:** Beata Jankowska-Polańska, Monika Czyrniańska, Kathie Sarzyńska, Natalia Świątoniowska-Lonc, Mariusz Chabowski

**Affiliations:** 1 Division of Nervous System Diseases, Department of Clinical Nursing, Faculty of Health Science, Wroclaw Medical University, Bartla Street 5, 51-618 Wroclaw, Poland; 2 Department of Paediatric Oncology, Haematology and Bone Marrow Transplantation, “Cape of Hope” Transregional Paediatric Oncology Centre, BorowskaStreet 213, 50-556 Wroclaw, Poland; 3 Internal Medicine Nursing Student Scientific Circles, Wroclaw Medical University, Bartla Street 5, 51-618 Wroclaw, Poland; 4 Department of Surgery, 4thMilitary Teaching Hospital, Weigla Street 5, 50-981 Wroclaw, Poland; 5 Division of Oncology and Palliative Care, Department of Clinical Nursing, Faculty of Health Science, Wroclaw Medical University, Bartla Street 5, 51-618 Wroclaw, Poland

**Keywords:** Chronic fatigue, Care rationing, Missed nursing care

## Abstract

**Background:**

Rationing of nursing care is a relatively new concept. It refers to an error of omission and has a direct influence on the quality of nursing care and treatment outcomes. Nurses who experience chronic fatigue often fail to perform their duties properly, which may lead, for instance, to medical errors attributed to impaired judgment. Therefore, it is necessary to identify factors which give rise to fatigue, leading to rationing of nursing care, and develop strategies to eliminate them. The primary objective of the study was to assess the impact of fatigue on nursing care rationing in paediatric haematology and oncology departments. The secondary objective of this study was to identify the factors, which may influence the nursing care rationing.

**Methods:**

The study was conducted among 95 nurses (aged between 23 and 58 years) workinginthe Department of Paediatric Oncology, Haematology and Bone Marrow Transplantation of the University Teaching Hospital in Wroclaw. Participation in the study was voluntary and anonymous. Our own sociodemographic questionnaire, the Basel Extent of Rationing of Nursing Carequestionnaire and the modified fatigue impact scale (MFIS) were used in the study.

**Results:**

The level of fatigue among the nurses participating in the study, as measured by the MFIS, was high, namely 28.97 ± 16.78. It was found that the fatigue of the nurses influenced most often the psycho-social dimension of QoL (1.78 ± 1.05), and least often - cognitive (1.24 ± 0.78). A correlation analysis showed that all aspects of fatigue had a statistically significant positive impact on care rationing (*p* < 0.05), i.e. the greater the fatigue, the higher the level of care rationing. A regression analysis showed that a 12-h shift pattern was an independent predictor of the level of care rationing (r = 0.771, *p* < 0.05).

**Conclusions:**

Nurses working in paediatric haematology departments report a high level of fatigue. Work pattern is an independent determinant of nursing carerationing. A high level of nursing care rationing was found for nurses working 12-h shifts.

**Trial registration:**

The study was approved by the Bioethics Committee of the Wroclaw Medical University, Poland (February 8th 2019, No. 205/2019).

## Background

The problem of nursing care rationing is being investigated around the world. In Poland, the number of nurses per 1000 population is still decreasing. This suggests that we are facing a problem of increasing nurse shortages [1]. The concept of missed nursing care was identified for the first time in 2006 by American nurse Beatrice J. Kalisch et al. [2]. Nursing care rationing takes place wheninsufficient resources are available to provide all patients with the care they require. However, one of many definitions, refers to rationing or disguised rationing in nursing care as the withholding or abandonment of necessary nursing measures for patients due to a lack of nursing resources, such as: staffing, different skills or time. The concept proposed by Schubert et al. defines rationing in terms of prioritization and decision-making, stressing that covert rationing occurs when nurses do not have enough time and resources to provide the holistic care they perceive their patients need [3].

Rationing is a process that is influenced by many factors relating to the patient and nurse, as well as the work environment, organizational resources, and care philosophy. All these elements influence the model of care. The model of loss of care or lack of continuity of care proposed by Kalisch et al. [4] emphasizes that deficiencies in nursing care are the result of external factors that create the environment and work style and are not dependent on nurses. The most frequest factors are: work pattern, job reductions, new treatment options, work environment, organisational resources and the patient himself. It is also linked to the problem of delayed or unfinished nursing care. A number of factors contribute to care rationing, including workload, job reductions, and fatigue, burn out,new treatment options, attitude of nurses (their knowledge, opinions and beliefs), work environment, organisational resources and the patient himself [3]. It is worth noting that there are numerous interactions between the above-mentioned factors.

Fatigue affects all the nurses and the rates of the nurse fatigue are increasing [5]. Nurses experienced a moderate to high level of acute fatigue and moderate levels of chronic fatigue and inter-shift recovery [6]. Polish nurses, multi-jobbing and the high patient-to-nurse ratio and given that nurses complain about being chronically fatigued and overworked. Many factors influence the nurse fatigue. These factors are: heavy workloads, staffing shortages, shift work, increased patient acuity, increased patient expectations, little time for professional development, decline in leadership, inadequate recovery time and personal factors and health-care cultures that create pressure for nurses to take on extra work [5–7]. Due to this, the impact of fatigue may be an important factor in rationing nursing care, especially in the group of nurses working with children with hematological disease. Moreover, numerous evidence and studies have indicated that overwork, fatigue and intense stress in the nursing profession can lead to a reduction in organizational commitment [8], which also affects care rationing [3].

Unfortunately, there is a lack of research in the literature on the rationing of nursing care in such specialised units as paediatric haematology and the factors influencing the rationing of nursing care. Despite the fact that fatigue is a common and well-researched topic in nursing, there are no studies that link fatigue with rationing. Therefore, the primary objective of the study was to assess the impact of fatigue on nursing care rationing in paediatric haematology and oncology departments. The secondary objective of this study was to identify the factors, which may influence the nursing care rationing.

## Methods

A cross-sectional study was conducted between February and May2019. The study included only registered nurses with a Polish nursing licence (BNS: bachelor’s, MNS: master’s degree in nursing sciences or secondary medical school) and a minimum of three years of work experience in direct patient care. The study included nurses working in the Department of Paediatric Oncology, Haematology and Bone Marrow Transplantation of the University Teaching Hospital in Wroclaw, Poland. The respondents cared for children (under the age of 18) with haematopoietic cancers. The exclusion criteria were: being a student of nursing and lack of consent to participate in the study.

107 nurses were initiallyqualified into the study. Five nurses did not meet the inclusion criteria, and 7 nurses did not give back the completed survey. Finally, a total of 95 nurses were included to participate in the study. Data were collected consecutively within a period of 4 months. All the nurses who met the inclusion criteria were invited to fill out the BERNCA-R questionnaires. The questionnaires were distributed by the ward head nurseand were collected in a closed box at the department.

The respondents were asked 16 questions included in our own questionnaire. The questions concerned basic socio-demographic data (age and sex) and work data (number of work years, number of work hours during the week, completed courses, number of patients in care during on-call duty, number of beds in the ward, number of hours at night during the week, education level, mode of work, number of jobs, satisfaction with earnings, reasons for dissatisfaction after overworked shift, performance of duties for other staff employed in the facility).

Moreover, two standardised questionnaires were used:

1. **Modified Fatigue Impact Scale (MFIS)** - it is used to assess the level of fatigue of respondents. The total MFIS score can range from 0 to 84. The higher the score, the greater the fatigue. There are no standards as to what scores indicate a high level of fatigue. The MFIS (Modified Fatigue Impact Scale) consists of three parts. Part 1 includes 9 questions assessing the perceived impact of fatigue on physical functions. Part 2 includes 10 questions about the impact of fatigue on cognitive functions. Part 3 includes two questions aimed to assess psychosocial functions. Respondents answer the closed questions in the questionnaire by indicating how often they have experienced the specific effects of fatigue. Answers are scored as follows: never – 1 point, rarely – 2, sometimes – 3, often – 4, and almost always – 5 points. The score on the first part of the questionnaire, which relates to physical functions, can range from 9 to 45, the score on the second part, which evaluates cognitive functions, can range from 10 to 50, and the score on the last part of the questionnaire, which evaluates psychosocial functions, can range from 2 to 10. The questionnaire has good psychometric properties and ithas been often used for the evaluation of the fatigue, which affects everyday life of patients. However, it has never been used for the evaluation how the fatigue influence the work of the nurse. Sensitivity and specificity for MFIS Total were 0.85 and 0.80, respectively; for MFIS Cognitive were 0.83 and 0.68, respectively; and for MFIS Physical/Activities 0.82 and 0.92, respectively. Cronbach α for all 21 items was 0.97, but for cognitive subscale was 0.95, and for Physical/Activities subscale was 0.96 [9].
2. **Basel Extent of Rationing of Nursing Care (BERNCA) questionnaire -** it is used to determine how often respondents ration nursing care (Table 4). It consists of questions about the frequency of 32 situations where nursing care rationing is necessary. It consists of 5 parts relating to such aspects as patients’ daily activities, care and support, rehabilitation and education, monitoring and safety as well as documentation. Respondents indicate how often in the past seven days they were unable to carry out the tasks listed in the questionnaire using a 5-point scale (0 = not required, 1 = never, 2 = rarely, 3 = sometimes, 4 = often). The higher the score, the higher the level of nursing care rationing [10]. Implicit rationing of nursing care was studied originally by Schubert et al. [10] in the context of the Rationing of Nursing Care in Switzerland (RICH) nursing study. In order to measure rationing of nursing care in that country, the researchers developed the Basel Extent of Rationing of Nursing Care (BERNCA) instrument. The initial validity (content and construct validity) and reliability of the BERNCA questionnaire were established using nurse survey data from five German-speaking Swiss hospitals [10]. Schubert et al. revised the BERNCA for the use in the Registered Nurse Forecasting (RN4CAST) study including a sample of 35 acute care hospitals from the German, French and Italian speaking regions of Switzerland [11]. Moreover study of Norman and Sjetne found good psychometric properties of the Norwegian version BERNCA-NH, assessed in a sample of care workers in Norwegian nursing homes [12]. The Polish version of the questionnaire was used, which had high internal consistency (range 0.1–0.84). Cronbach’s alpha for the unidimensional scale was 0.96 [13].

The study was approved by the Bioethics Committee of the Wroclaw Medical University, Poland (February 8th 2019, No. 205/2019). All subjects were informed about the purpose and conduct of the study and gave written informed consent to participate in the study.

### Statistical analysis – method description

Quantitative variables (such as BERNCA and MFIS results, age, working experience) were compared between two groups using Mann-Whitney test and between three or more groups using Kruskal-Wallis test. Correlations between quantitative variables were analysed using Spearman’s correlation coefficient [14].

A multivariate analysis of the independent correlations of multiple variables on a qualitative variable was performed using linear regression. The results are presented as the values of parameters of the regression model with a 95% confidence interval. The normality of the distribution of variables was verified using the Shapiro-Wilk test. A level of significance of 0.05 was used in the analysis. Thus, all *p* values of less than 0.05 were interpreted as indicating significant correlations. The analysis was performed using the R software, version 3.4.3 [15]..

## Results

The average age of the respondents was 37, whereas their average work experience was around 15 years. The vast majority of the respondents were women (98%). Of the respondents, 67% had no specialist qualifications. The respondents worked 46 h a weekon average, including around 20 h at night. The average number of beds in the department was 16 and the average number of patients per nurse was 8.

The respondents were also asked about their education. The largest proportion of the respondents had a Bachelor’s degree (41%), 28% had a Master’s degree, whereas 25% had graduated from a secondary medical school. The majority of the respondents worked 12-h shifts (82%) and had only one job (77%). Almost all the respondents declared that they performed the duties of other staff working at the hospital, with the majority (59%) declaring that they performed the duties of a physician. As many as 88% of the respondents considered their salary to be unsatisfactory. The information discussed above is shown in Table 1 and Table 2.

**Table 1:** Means for selected variables

Variable	Mean (SD)	Median (quartiles)
Age [years]	37.43 (10.9)	40 (26–45,5)
Work experience [years]	14.96 (11.7)	17 (3–24,5)
Number of courses completed	3.55 (2.65)	3 (2–5)
Weekly working hours	46.58 (9.21)	48 (40–48)
Number of hours worked at night per week	20.4 (9.67)	24 (14–24)
Number of beds in the department	16.12 (9.96)	14 (10–20)
Number of patients per nurse during a day	8.04 (7.81)	6 (3–8)
Distance from home to work [km]	17.22 (14.5)	11 (5–30)

SD – standard deviation.

**Table 2:** Selected characteristics of the respondents, expressed as numbers and percentages

Variable		N	%
Sex	Female	93	97.90
	Male	2	2.11
Education	Secondary medical school	24	25.26
	Post-secondary medical school	5	5.26
	Bachelor’s degree	39	41.05
	Master’s degree	27	28.42
	Doctor’s degree	0	0.00
Number of specialisation courses completed	None	64	67.37
	1	29	30.53
	2	2	2.11
Work pattern	8-h shifts	14	14.74
	12-h shifts	79	83.16
	24-h shifts	2	2.11
Number of jobs held	1	73	76.84
	2	21	22.11
	3	1	1.05
Performance of the duties of other medical staff?	Physician	56	58.95
	Ward attendant	42	44.21
	Physiotherapist	16	16.84
	Orderly	55	57.89
	Never	24	25.26
Satisfaction with salary (one job)	Yes	11	11.58
	No	84	88.42
Dissatisfaction after a shift - reasons:	Failure to perform all duties	65	68.42
	Exhaustion	92	96.84
	Lack of clarity with regard to job roles	57	60.00
	Unclear recommendations, tasks beyond one’s competence	62	65.26

N –- number of people;

Mean point result of BERNCA questionnaire (*n* = 95) was 1.61 ± 0.85.Individual people did not answer some of the questions: 1 person - questions 4, 7, 9 and 30; and two people - question 12.The analysis of responses to particular questions in the BERNCA questionnaire showed thatmore than half of the respondents had never been unable to perform adequate hand hygiene (51%) and disinfect their hands adequately (52%). Unfortunately, one-fourth of the respondents reported that they sometimes had not had enough time to inform themselves about the condition of particular patients and study their care plans at the beginning of their shift. Thirty-two percent had rarely been unable to assess the needs of newly admitted patients, 29%declared that they had rarely been unable to set up patients’ care plans and 34% had not been able to document and evaluate the performed nursing care for a patient in a timely manner. Twenty-eight percent of the respondents declared that they had not needed to ration dental hygienetasks, and the same percentage reported that they had never omitted those tasks. As many as 31% of the respondents had not needed to omit tasks related to assisting patients unable to eat independentlyand the same percentage stated that they had not needed to ration tasks related to mobilising or changing the position of patients who were immobile A similar percentage (29%) of participants reported that they had sometimes been unable to administer a prescribed medication or infusion at the recommended. Thirty three % of the respondents had sometimes been unable to monitor patients as closely as had been prescribed by their physicians and 27% had sometimes been unable to monitor patients as closely as they felt was necessary. But on the other handa high number of the respondents (45%) did not need to ration tasks related to necessary sponge baths, and as many as 42% reported that they did not need to omit tasks related to partial sponge baths. About33%of the respondents reported that they had never delayed changing soiled linen or informing patients about planned tests. Twenty-nine percent of the respondents had never rationed tasks related to skin care and oral hygiene the same percentage of responders had rarely been unable to offer emotional or psychosocial support to a patient and 30% declared that had rarely been unable to have a necessary conversation with a patient or his / her family. Twenty-six percent of the respondents had never rationed tasks related to preparing patients for hospital discharge. Twenty-nine percent of the respondents reported that they had never had to delay necessary measures to assist patients with unforeseen sudden changes in status because the physicians called took a long time to arrive. In turn, 35% of the respondents reported that they had never delayed changing a wound dressing for a patient as needed, and 39% had never omitted tasks relating to preparing patients for tests. Thirty-six percent declared that they had never kept a patient who had rung for a nurse waiting longer than 5 min. (Table 3).

**Table 3:** Percentage distribution of responses to particular questions in the BERNCA questionnaire

QUESTIONS How often in the last 7 work days did it happened that:	% OF RESPONCES					
	Not required	Never	Rarely	Sometimes	Often	Mean point per question ± SD
1you could not assist a patient with a necessary sponge bath?	45.26	23.16	13.68	13.68	4.21	1.08 ± 1.23
2. you could not assist a patient with a necessary partial sponge bath?	42.11	25.26	13.68	14.74	4.21	1.14 ± 1.23
3. you could not carry out necessary skin care for patients?	22.11	29.47	25.26	13.68	9.47	1.59 ± 1.24
4. you could not carry out necessary oral hygiene for patients?	24.21	29.47	23.16	12.63	10.53	1.56 ± 1.28
5. you could not carry out necessary dental hygiene for patients?	28.42	28.42	21.05	11.58	10.53	1.47 ± 1.3
6. you could not appropriately assist patients unable to eat independently?	31.58	26.32	18.95	18.95	4.21	1.38 ± 1.23
7. you were unable to mobilise patients who had restricted mobility or who were immobile?	30.85	26.6	23.4	11.7	7.45	1.38 ± 1.25
8. you were unable to change the position of patients who had restricted mobility or who were immobile?	30.53	26.32	18.95	17.89	6.32	1.43 ± 1.27
9. you could not change, in an adequate time period, patients’ bed linen soiled with urine, stool or vomit?	17.02	32.98	23.4	19.15	7.45	1.67 ± 1.19
10. you could not offer emotional or psychosocial support to a patient, even though you felt it was necessary, e.g. dealing with insecurities and fear of his / her illness, the feeling of dependency?	9.47	26.32	29.47	23.16	11.58	2.01 ± 1.16
11. you could not have a necessary conversation with a patient or his / her family?	7.37	27.37	30.53	23.16	11.58	2.04 ± 1.13
12. you could not inform patients sufficiently about planned tests or therapies?	9.68	33.33	20.43	24.73	11.83	1.96 ± 1.21
13. you did not have time for toilet or continence training and therefore had to put a patient in diapers?	34.74	26.32	20	12.63	6.32	1.29 ± 1.25
14. you did not have time for toilet or continence training and therefore had to insert a catheter?	37.89	31.58	10.53	12.63	7.37	1.20 ± 1.28
15. you could not carry out activating or rehabilitating care for patients?	36.84	23.16	14.74	16.84	8.42	1.37 ± 1.35
16. you were unable to provide patients and / or their families with training, e.g. on insulin injection or coping with illness-specific symptoms (hypoglycaemia, dyspnoea)?	42.11	24.21	16.84	13.68	3.16	1.12 ± 1.19
17. you were unable to fully prepare patients or their families for hospital discharge?	21.05	26.32	24.21	23.16	5.26	1.65 ± 1.2
18. you were unable to monitor patients as closely as had been prescribed by their physicians?	6.32	31.58	20	33.68	8.42	2.06 ± 1.12
19. you were unable to monitor patients as closely as you felt was necessary?	9.47	29.47	24.21	27.37	9.47	1.98 ± 1.16
20. you were unable to watch confused patients carefully enough and therefore had to restrain them?	42.11	23.16	17.89	12.63	4.21	1.14 ± 1.22
21. you were unable to watch confused patients carefully enough and therefore had to sedate them?	42.11	27.37	14.74	11.58	4.21	1.08 ± 1.19
22. you were forced to delay necessary measures to assist patients with unforeseen sudden or acute changes in status because the physicians called took a long time to arrive?	21.05	29.47	23.16	20	6.32	1.61 ± 1.21
23. you were unable to administer a prescribed medication and / or infusion at the recommended time?	8.42	27.37	27.37	29.47	7.37	2 ± 1.1
24. you could not change/apply a wound dressing for a patient as needed?	10.53	34.74	20	29.47	5.26	1.84 ± 1.12
25. you could not prepare patients for planned tests or therapies?	11.58	38.95	25.26	20	4.21	1.66 ± 1.06
26. you had to keep a patient who had rung for a nurse waiting longer than 5 min?	16.84	35.79	23.16	13.68	10.53	1.65 ± 1.22
27. you could not perform adequate hand hygiene?	7.37	50.53	16.84	21.05	4.21	1.64 ± 1.03
28. you could not disinfect your hands adequately?	8.42	51.58	16.84	20	3.16	1.58 ± 1.01
29. you did not have enough time to inform yourself about the condition of particular patients and study their care plans at the beginning of your shift?	10.53	26.32	27.37	25.26	10.53	1.99 ± 1.17
30. you were unable to properly assess the needs of newly admitted patients?	9.57	22.34	31.91	25.53	10.64	2.05 ± 1.14
31. you were unable to set up patients’ care plans?	11.58	25.26	29.47	24.21	9.47	1.95 ± 1.16
32. you could not adequately document and evaluate the performed nursing care for a patient?	6.32	28.42	26.32	33.68	5.26	2.03 ± 1.05

SD – standard deviation

The analysis of the BERNCA questionnaire results showed that the most frequently rationed nursing interventions included monitoring patients as closely as had been prescribed by their physicians (2.06 ± 1.12), assessing the needs of newly admitted patients (2.05 ± 1.14), documenting and evaluating the performed nursing care for a patient (2.03 ± 1.05), having a necessary conversation with a patient or his / her family (2.04 ± 1.13) and providing support to patients (2.01 ± 1.16).

In turn, the lowest level of rationing was found for such items of the questionnaire as restraining confused patients due to the inability to watch them carefully enough (1.14 ± 1.22), assisting a patient with a necessary sponge bath (1.08 ± 1.23) or a necessary partial sponge bath (1.14 ± 1.23) as well as sedating confused patients due to the inability to watch them carefully enough (1.08 ± 1.19).

The average MFIS score of the respondents was 28.97 ± 16.78. It was found that u badanych zmęczenie najczęściej miało wpływ na psychosocial wymiar jakości życia1.78 ± 1,05a najrzadziej na ognitive 1.24 ± 0,78.(Table 4).

**Table 4:** Scores for fatigue level on particular subscales of the MFIS

MFIS subscale (range of points)	N	Subscale mean score (Me±SD)	Question mean score (Me±SD)	Median	Range (min – max)
Physical (0–36)	95	12.97 ± 7.53	1.44 ± 0,84	12	1–32
Cognitive (0–40)	95	12.43 ± 7.76	1.24 ± 0,78	12	0–36
Psychosocial (0–8)	95	3.57 ± 2.1	1.78 ± 1,05	3	0–8
MFIS – total score	95	28.97 ± 16.78	**–**	28	1–75

Me – mean; SD – standard deviation; N –- number of people; MFIS - Modified Fatigue Impact Scale

### Correlations of selected variables on rationing

The study did not show a statistically significant correlations of age, work experience and the number of patients per nurse on care rationing (*p* > 0.05) (Table 5).

**Table 5:** The results of correlation analysisbetween selected variables and the level of care rationing(BERNCA)

Variables	Correlation coefficient	p value
Age and care rationing	0.065	0.531
Work experience and care rationing	0.021	0.842
Number of patients per nurse and care rationing	0.165	0.111

p - probability value

The analysis of the correlations of such variableswith rationing as the number of jobs held by a nurse, work pattern, education and satisfaction with salary on nursing care rationing showed that work pattern was the only determinant of the nursing care rationing level (*p* < 0.05). Other variables such as the number of jobs held, education and job satisfaction did not have a statistically significant impact on nursing care rationing (*p* > 0.05). **(**Table 6**).**

**Table 6:** The results of correlation analysis of selected variables with nursing carerationing

Variable	N	Mean	SD	Median	Range (min-max)	p
1 job	73	1.62	0.84	1.47	0–3.94	0.993
2–3 jobs	22	1.6	0.91	1.47	0–3.22	
8-h shifts	14	0.83	0.56	0.88	0–1.88	**< 0.001**
12-h shifts	78	1.72	0.82	1.58	0,16–3.94	
High school education	29	1.58	0.87	1.47	0–3.94	0.822
Bachelor’s degree	39	1.68	0.92	1.56	0–3.56	
Master’s degree	27	1.55	0.75	1.47	0,38–3.22	
Satisfactory salary	11	1.38	0.66	1.25	0,53–2.84	0.346
Unsatisfactory salary	84	1.64	0.87	1.55	0–3.94	

p - probability value; SD – standard deviation; N –- number of people; BERNCA–BaselExtent of Rationing of Nursing Care

### Rationing of nursing care and fatigue

The total MFIS score was positively correlated with the BERNCA score, which means that the greater the fatigue, the higher the level of care rationing. A correlation analysis showed a weak positive correlation for all the subscales of the MFIS questionnaire– physical: r = 0.496, *p* < 0.001; cognitive: r = 0.481, p < 0.001; psychosocial: r = 0.332, *p* = 0.001 (Table 7).

**Table 7:** The results of correlation analysisbetween particular subscales of the fatigue questionnaire (MFIS) and care rationing (BERNCA)

MFIS	Correlation with the BERNCA score			
	Correlation coefficient	p	Direction	Strength
Total score	0.486	< 0.001	positive	weak
Physical subscale	0.496	< 0.001	positive	weak
Cognitive subscale	0.481	< 0.001	positive	weak
Psychosocial subscale	0.332	0.001	positive	weak

MFIS - Modified Fatigue Impact Scale; p - probability value; BERNCA–BaselExtent of Rationing of NursingCare

### The analysis of impact regression ofselected variables with nursing carerationing (BERNCA)

A linear regression model showed that work pattern is an independent predictor of the care rationing level (*p* < 0.05). When compared with an 8-h shift pattern, a 12-h shift pattern increased the BERNCA score by an average of 0.771 points, which means that the level of nursing care rationing is higher in the case of nurses working 12-h shifts than in the case of nurses working 8-h shifts (Table 8).

**Table 8:** **The results of impact regression of** selected variables with nursing care rationing (BERNCA)

Variable		Regression parameter	95% CI		p
Age [years]		0.049	−0.017	0.115	0.144
Work experience [years]		−0.036	− 0.096	0.025	0.245
Number of patients per nurse during a day		0.02	−0.001	0.042	0.063
Distance from home to work [km]		0.008	−0.003	0.019	0.135
MFIS: Physical subscale		0.044	−0.02	0.109	0.176
MFIS: Cognitive subscale		0.015	−0.041	0.071	0.595
MFIS: Psychosocial subscale		−0.056	−0.186	0.074	0.395
Number of jobs held	1	ref.			
	2–3	−0.002	− 0.353	0.35	0.992
Work pattern	8-h shifts	ref.			
	12-h shifts	0.771	0.359	1.184	**< 0.001**
Education	High school	ref.			
	Bachelor’s degree	0.338	−0.099	0.774	0.128
	Master’s degree	0.435	−0.028	0.899	0.065
Satisfaction with salary	Satisfactory	ref.			
	Unsatisfactory	0.314	−0.211	0.839	0.238

CI – Confidenceinterval; p - probability value

## Discussion

The present paper - as one of the few - focuses on the important topic of the fatigue which influence the nursing care rationing. Nowadays, missed care is a common threat, as we face a global shortage of nurses. Therefore, this problem should be more thoroughly addressed, since it can result in potentially dangerous medical errors. Recognising that the work environment of nurses has an impact on treatment outcomes for patients, we should attempt to develop the best conditions for nurses to provide them with a satisfying job.

In our study, the lowest level of care rationing was observed for activities related to the direct care of patients, including devoting sufficient time to patients and assisting them with a necessary sponge bath or partial sponge bath. In turn, the activities identified as being most frequently rationed were those associated with documentation and provision of emotional support, including monitoring the condition of patients, assessing the needs of newly admitted patients, documenting and evaluating the performed nursing care for a patient, having a conversation with a patient and providing emotional support to patients and their families. The findings from our study are to a significant extent consistent with the study on Norwegian nurses carried out by Norman et al. in 2019 [12]. Our findings are also consistent with the study by Schubert et al. of 2010, in which the highest level of care rationing was reported for similar interventions as those listed above [16].

In turn, a study by Henderson et al. reported the highest level of care rationing for unplanned activities (e.g. answering call bells) and those relating to rehabilitative care, which was not confirmed in our study [17].

A study by Schubert et al. on 1633 nurses from 12 hospitals in Europe found that the most frequently rationed activities included documenting the care provided to patients, setting up and getting acquainted with nursing care plans, monitoring, and providing emotional support and having a necessary conversation with a patient, which is consistent with the findings from our study. Contrary to our findings, the aforementioned study found that such interventions as the provision of information on therapies and administration of a medication / infusion at the recommended time, were frequently rationed, too. In turn, the study showed that the least frequently rationed tasks were those associated with care routines and the daily functioning of patients, which is consistent with the results of our study [11].

Zúñiga et al. reported surprising findings from their study on Swiss nurses working in nursing homes. They found that tasks directly relating to care routines and assistance with daily activities were among the most frequently rationed nursing interventions. In turn, the study showed that the least frequently rationed activities included all tasks related to the documentation of the care provided to patients and those associated with patients’ nursing care plans [18]. On the other hand, in a study conducted among German nurses (34%) and our own research (33%), there is an overlap in only one activity most frequently subject to rationing, namely checking the condition of the patient as often as required [16]. There was also no relationship between our study and the results obtained by Schubert, where the following activities were subject to rationing: emotional support, conversation with the patient or his/her family and development of a care plan. The only point in common between the two studies was only one nursing task least frequently omitted, i.e.: changing soiled bed linen [19]. It is worth pointing out that 51% of the respondents in our study reported that they had never omitted washing and disinfecting hands, which is consistent with the results of the study by Schubert et al. [11]. In turn, another study by Schubert et al. found no correlation between the number of patients per nurse and increased level of rationing of care, which is consistent with the findings from our study [19].

Kalisch et al. reported interesting findings from their study on 459 nurses in 3 hospitals, whereas many as 79% of the study participants declared that they had been unable to fully document the care provided to patients [20]. In turn, a study by Norman et al. on care workers in 162 nursing home units in different parts of Norway found that the documentation and evaluation of interventions were among the most frequently rationed activities [12]. The study by Zúñiga found that more rationing of documentation was associated with better quality of care. Rationing of documentation allows care workers to spend more time on other activities that are seen as more important in terms of the quality of care [18]. The differences between the survey results of Norwegian nurses and those of Polish nurses may be explained by differences in terms of workplaces and specificity of care. Patients in Norwegian care homes are elderly and many of them have moderate to severe physical limitations. They believe that their psychological and social needs are more important and meaningful than activities that maintain their physical abilities [21]. In the case of the residents of care homes, psychosocial care plays a key role in optimising treatment outcomes, including well-being, independence and healing.

Longer work hours, overtime, working night shifts, and rotating shift work were found to be associated with increased fatigue and an increased risk of medical errors or near-misses [22]. Our study confirmed a correlation between all the fatigue subscales and nursing carerationing. There is scientific evidence that chronic fatigue syndrome has an impact on nursing carerationing, i.e. the omission (which does not pertain to occasional situations or when the life of a patient is in danger) of care relating to: mobilising patients, changing their position, feeding, education, discharge plans, provision of emotional support, hygiene, acceptance and provision of documentation and observation [23]. According to a report by the Central Institute for Labour Protection - National Research Institute, shift work has an impact on the level of fatigue (night-shift work contributes to chronic external desynchronization). Whenever a shift pattern changes (e.g. from a morning shift to a night shift, or vice versa), an adjustment process takes place, which was not confirmed in our study [24]. In turn, according to the literature and the findings from our study, other demographic variables such as age and level of education, are not correlated with the level of fatigue [25]. However, a study by Chojnacka-Szawłowska confirmed that chronic fatigue increases with age [26]. In a study by Yoder, a greater level of chronic fatigue was reported for nurses working 8-h shifts compared to those working 12-h shifts [27], which is not consistent with the findings from our study or a study by Kułagowska and Kosińska, who found that a 12-h shift pattern is associated with greater fatigue [28]. Similarly, a study by Mirzaei et al. showed that 12-h shifts, both day-time and night-time, were associated with a greater mental strain, which was directly correlated with chronic fatigue [29].

According to the literature, factors correlated with the level of nursing care rationing include night-shift work and age (younger nurses). This was not confirmed in our study, which found that age was not correlated with the level of rationing [30]. In turn, the study by Kalisch et al. conducted in 2011 found that the age of nurses did not have a correlation with care rationing. The same study demonstrated that the higher the number of patients per nurse, the higher the level of care-rationing. However, our study did not confirm such a correlation [31]. In turn, according to Dutra et al., the main causes of missed care include insufficient staff resources, in particular a shortage of specialists, urgent situations with patients during a shift and unavailability of necessary medications, materials or equipment [32].

We found that a shift work pattern, namely 8-h and 12-h shifts, was the only statistically significant independent variable that had statistical significant correlation (*p* < 0.05) with care rationing. Nurses working 12-h shifts rationed care more frequently compared with nurses working 8-h shifts. The findings are confirmed by other studies, which found that nurses working 10-h shifts or longer were up to two-and-a-half times more likely to experience burnout and job dissatisfaction, and to intend to leave the job compared with nurses working shorter shifts. Moreover, it was demonstrated that shift work had a negative effect on the well-being of nurses and might be detrimental to the care of patients [33]. Bollschweiler et al. proved that the quality of patient care (recovery, mortality and duration of stay in hospital) was significantly better when care workers worked 12-h shifts vs. 8-h shifts [34]. In turn, when analysing the patterns of nursing care, Reid et al. found that nurses working 12-h shifts provided lower quality patient care compared with nurses working 8-h shifts [35]. Rogers et al. found that the likelihood of making an error was three times higher when nurses worked at least 12.5 consecutive hours than when they worked shorter shifts [36]. Similarly, Scott et al. found that the likelihood of making an error was almost two times higher when nurses worked shifts lasting 12.5 h or more [37].

The present research had some limitations. The one of them is the convenient nature of the study group. Another limitation was the lack of answers from the participants of this research. However, the questions that the respondents did not answer were taken into account and did not affect the statistical results. Although the results of the study clearly showed a positive correlation between the fatigue of nurses of pediatric hematology and the rationing and the relationship between shift work and the above-mentioned concepts, due to the small number of respondents, they cannot be generalized to the entire nursing group. However, the results may be valuable for further research to identify the unknown factors influencing rationing nursing care. This is very important to implement the new programs in medical facilities that would allow less rationing of nursing care. The effectiveness of these activities is possible only thanks to the knowledge of all the factors that influence the rationing of nursing care.

Our study has several limitations. The first is the use of a standardized Fatigue Impact Assessment Tool (MFIS) that has not been used in nursing studies so far. Another limitation is the selection of the study group only from one center. In the future, it would be worthwhile to compare the results of the study between several centers and conduct them in other departments. The virtue of this study is the fact that it is the first study in Poland and one of the few that assesses the impact of fatigue on rationing nursing care. According to our survey, employers should consider making a changefrom a 12-h shift pattern to 8-h shiftsinorder to identify more determinants of nursing care-rationing, a study on a larger group of nurses should be carried out to verify whether a shift work pattern is really the only factor that shows the correlation with rationing of care by Polish nurses. It may also be interesting to consider the workshops for nurses in order to teach them how to regulate their stress, restore their energy, and perform self-care. It will not only benefit the nursing profession, but also - the patient care, which was underlined by Landro et al. [38]. Further research is necessary to identify the potential causes of rationing in order to counter the aforementioned effects of an “error of omission” in nursing and develop a plan for the elimination of factors that significantly affect the quality of nursing care given the high workload.

## Conclusions

12-h shift pattern is the independent determinant of nursing care rationing.

The fatigue most often influenced the psychosocial, and least often - the cognitive dimension of the QoL of the nurses.

## Data Availability

The datasets supporting the conclusions of this article are included within the article.

## Abbreviations

BERNCA: Basel Extent of Rationing of Nursing Care
CI: Confidence interval
Me: mean
MFIS: Modified Fatigue Impact Scale
N: number of people
p: probability value
SD: standard deviation
